# Candidate genes and gene markers for the resistance to porcine pleuropneumonia

**DOI:** 10.1007/s00335-019-09825-0

**Published:** 2020-01-20

**Authors:** Florian Nietfeld, Doris Höltig, Hermann Willems, Peter Valentin-Weigand, Christine Wurmser, Karl-Heinz Waldmann, Ruedi Fries, Gerald Reiner

**Affiliations:** 1 Department for Veterinary Clinical Sciences, Justus-Liebig-University, Giessen, Germany; 2 Clinic for Swine, Small Ruminants, Forensic Medicine and Ambulatory Service, University of Veterinary Medicine Hannover, Foundation, Hannover, Germany; 3 Institute for Microbiology, University of Veterinary Medicine Hannover, Foundation, Hannover, Germany; 4 Chair of Animal Breeding, Technical University of Munich, Freising, Germany

## Abstract

*Actinobacillus* (*A*.) *pleuropneumoniae* is one of the most important respiratory pathogens in global pig production. Antimicrobial treatment and vaccination provide only limited protection, but genetic disease resistance is a very promising alternative for sustainable prophylaxis. Previous studies have discovered multiple QTL that may explain up to 30% of phenotypic variance. Based on these findings, the aim of the present study was to use genomic sequencing to identify genetic markers for resistance to pleuropneumonia in a segregating commercial German Landrace line. 163 pigs were infected with *A. pleuropneumoniae* Serotype 7 through a standardized aerosol infection method. Phenotypes were accurately defined on a clinical, pathological and microbiological basis. The 58 pigs with the most extreme phenotypes were genotyped by sequencing (next-generation sequencing). SNPs were used in a genome-wide association study. The study identified genome-wide associated SNPs on three chromosomes, two of which were chromosomes of QTL which had been mapped in a recent experiment. Each variant explained up to 20% of the total phenotypic variance. Combined, the three variants explained 52.8% of the variance. The SNPs are located in genes involved in the pathomechanism of pleuropneumonia. This study confirms the genetic background for the host’s resistance to pleuropneumonia and indicates a potential role of three candidates on SSC2, SSC12 and SSC15. Favorable gene variants are segregating in commercial populations. Further work is needed to verify the results in a controlled study and to identify the functional QTN.

**Electronic supplementary material:**

The online version of this article (10.1007/s00335-019-09825-0) contains supplementary material, which is available to authorized users.

## Introduction

The pathogen *Actinobacillus (A.) pleuropneumoniae* is a gram-negative bacterium from the genus *Pasteurellaceae* and one of the major bacterial pulmonary pathogens in swine (Gottschalk 2012). The course of disease ranges from peracute, acute and subacute to chronic. Symptoms include high fever, dyspnoea, cyanosis and foamy blood-tinged discharge from mouth and nostrils. Sudden death can occur in fattening pigs. Adhesive pleuropneumonia is predominantly found in the slaughterhouse, whereas the severe clinical progression causes hemorrhagic necrotizing pleuropneumonia (Haesebrouck et al. 1997). Therefore, and due to the high losses, *A. pleuropneumoniae* is an important factor in pig production worldwide and needs to be effectively controlled (Losinger 2005).

*Actinobacillus pleuropneumoniae* is transmitted from sow to piglets. The main problem with a vaccination strategy appears to be a low cross-immunity between serotypes (Higgins et al. 1985; Fenwick and Henry 1994). In addition, modern subunit or toxoid vaccines do not provide full protection against the clinical outbreak of the disease (e.g., reduction in mortality, increase in daily weight gain, amounts of condemnations for pneumonia) (Chiers et al. 1998; Sjölund and Wallgren 2010; Jirawattanapong et al. 2010; Del Pozo Sacristan et al. 2014). Thus, antibiotics still prevail as the treatment of choice. However, increased application of antibiotics encourages the development of antibiotic resistance (White et al. 2002; Michael et al. 2018), which is not compatible with the demand for residue-free pork. Additionally, *A. pleuropneumoniae* provides major obstacles to the freedom of pigs from pain, suffering and damage (Reiner 2009).

One possible and sustainable solution could be to apply natural disease resistance or resilience (Davies et al. 2009). Evidence of host genetic variation with regard to resistance or tolerance has been described for more than 50 diseases in many economically important livestock species (Bishop and Woolliams 2014).

Resistance to *A. pleuropneumoniae* has been described by different authors and in different pig breeds (Straw et al. 1983; Jones 1986; Hoeltig et al. 2009). Differences between lines of German Landrace and Hampshire pigs were used to map QTL for resistance to *A. pleuropneumoniae* in a F2 family, with the highest effects on SSC2 and SSC12 (Reiner et al. 2014a), and to show associations between resistance and a broad range of differentially expressed genes (Reiner et al. 2014b). The QTL identified in this study were in good agreement with those mapped in a study of slaughter pigs (Gregersen et al. 2010).

The aim of the present study was to refine QTL mapping (Reiner et al. 2014a) by using a higher marker density by genotyping by sequencing (GBS) and more informative meioses in a segregating commercial pig population (German Landrace) in order to provide gene markers for the selection of more resistant pigs and to generate new insights into the pathogenesis of pleuropneumonia.

## Materials and methods

### Experimental animals

In this study, 163 pigs were experimentally infected with *A. pleuropneumoniae* at age 7 weeks. The animals belonged to a nucleus herd of the German Landrace and arrived 3 weeks before infection. They were vaccinated against *Mycoplasma hyopneumoniae* and Porcine Circovirus Type 2 (PCV-2). The accommodation accorded with guidelines for protection of vertebrate animals used for experimental and other scientific purposes, European Treaty Series, nos. 123 /170 (https://rm.coe.int/168007a67b). The Commission for Ethical Estimation of Animal Research Studies of the Lower Saxonian State Office for Consumer Protection and Food Safety (approval number: 33.12-42502-04-15/1962) approved the study terms.

Eight to ten pigs were kept together in pens with an area of 8 square meters. They were fed a commercial standardized diet (energy: 7–11 MJ/ME/day/piglet; protein: 90-165 g/day/piglet, increasing with age and bodyweight).

### Clinical examination and infection

Before infection, all pigs underwent a clinical, radiographic and sonographic examination as described in the study by Hoeltig et al. (2009). The radiographic examination was performed in latero-lateral and dorso-ventral view with a film focus distance of 1.5 m. Bronchoalveolar lavage fluids (100 ml 0.9% NaCl solution ad us. Vet., Co. WDT, Garbsen, Germany) and serum samples were taken. Bronchoalveolar lavage fluids were examined for respiratory pathogens and for the absence of *A. pleuropneumoniae* using bacterial culture and PCR, as described previously (Hoeltig et al. 2009). Serum samples were used for DNA extraction and antibody analysis via an ApxIV-Elisa (IDEXX APP—Apx-IV Ab Test®, Co. IDEXX Laboratories, Maine, USA).

Radiographic and sonographic examination as well as the extraction of bronchoalveolar lavage fluids were performed under general anesthesia with 20 mg/kg ketamine (Ketamine 100 mg/ml®, Co. CP-Pharma, Burgdorf, Germany) and 2 mg/kg azaperone (Stresnil®, Co. Janssen-Cliag GmbH, Baar, Switzerland) via intramuscular injection.

The aerosol infection was designed to last 30 min (Jacobsen et al. 1996). Approximately 1 × 10^5^ bacteria (*A. pleuropneumoniae* serotype 7, AP76*)* were nebulized to achieve an aerosol concentration of 1 × 10^2^ colony forming units per liter aerosol. Five to six pigs were infected simultaneously in a specially designed infection chamber.

Forty-eight hours post infection (p.i.), pigs were clinically monitored twice a day in a 2 h interval until day 7 p.i. Clinical examination consisted of evaluating each pig’s general appearance with relation to feed intake, general posture, temperature, vomiting, and a detailed examination of the respiratory tract. For a more detailed description of the procedure, see Hoeltig et al. (2009). Rescue criteria were defined analogous to the study by Hoeltig et al. (2009). The event of death between infection and necropsy was recorded.

### Pathological examination

After day 7 p.i. a further sonographic, radiographic and endoscopic examination was performed analogous to the examination three weeks before infection. Subsequently, the animals were euthanized by intravenous application of 80 mg/kg bodyweight pentobarbital (Euthadorm®, Co. cp-pharma, Grossburgwedel, Germany), and pathologically examined. Each non-physiological part was marked in the schematic map of the seven lung lobes. Lung lobes were then subdivided into 32 parts and scored according to the system by Hannan et al. (1982). For more details, see Hoeltig et al. (2009) and Reiner et al. (2014a, b).

### Bacteriological examination

Tissue samples for bacteriological examination were taken from each of the seven lung lobes as well as the tonsils and one lung lymph node to apply the semi-quantifying reisolation score (RIS) (Maas et al. 2006b). The tissue was cultured on modified Columbia agar (Jacobsen and Nielsen 1995) and score points were given from 0 (no growth of *A. pleuropneumoniae*) to 3 (growth in the swabbed area as well as in both fractionated streaks).

### Selection of pigs for sequencing

**Fig. 1:**
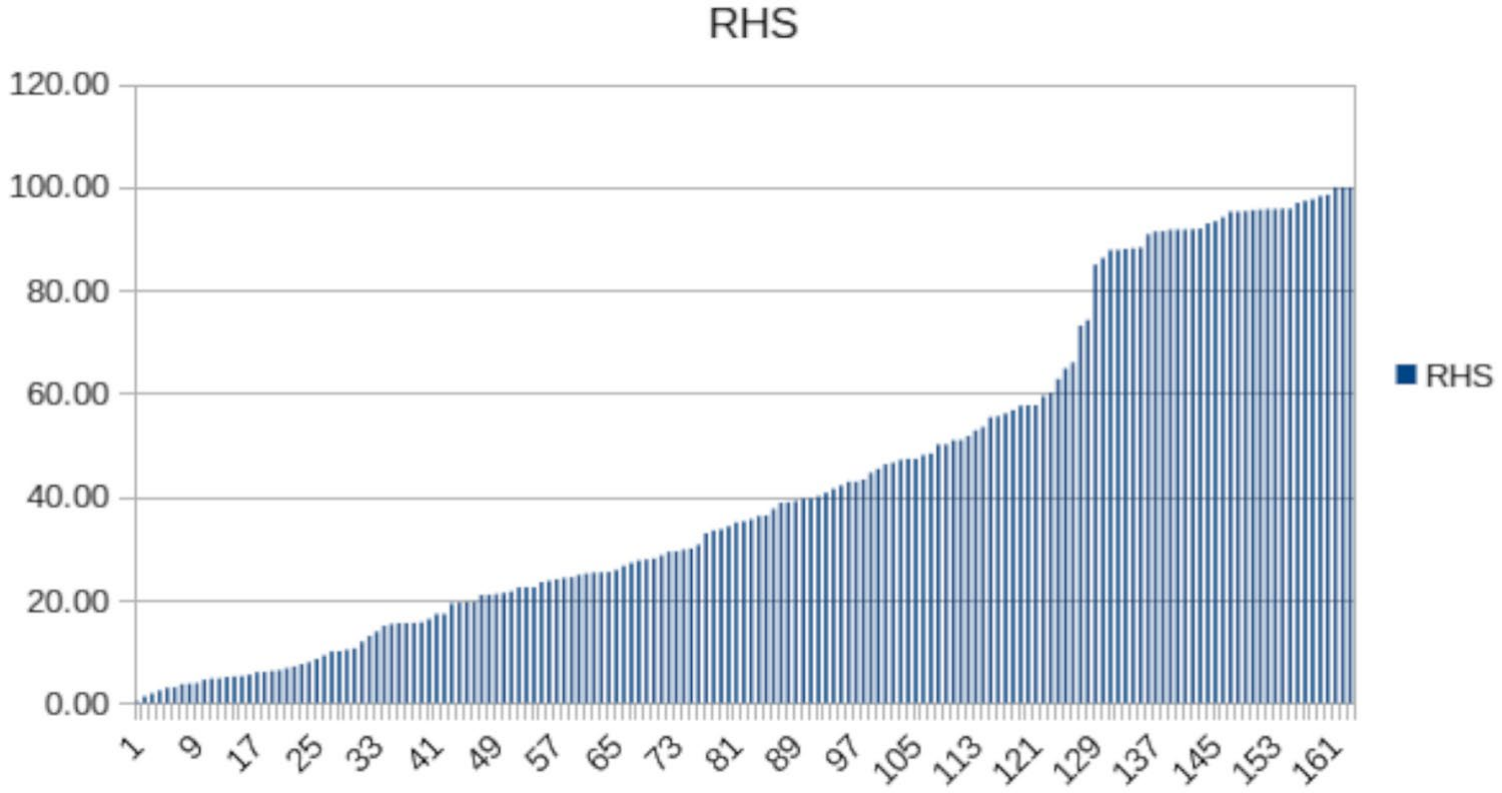
The RHS score increased steadily until animal no. 122, thereafter abruptly until animal no. 129 and finally reached constant high values between 85 and 100 (created with Excel)

Sorting the pigs according to their respiratory health score (RHS) in ascending order, there were several smaller inflection points in the rise of the curve (Fig. 1). Between animals 126 and 129, the RHS rose from 66 to 85 and then asymptotically approached the 100% limit. From this group of most sensitive animals, the 37 pigs with highest scores were selected for sequencing. Selecting the most resistant animals was less simple. The transitions between pig 1 (lowest RHS) and pig 126 were very gradual, with no threshold between resistant and intermediate types. Only some smaller inflection points were visible. In order to exclude intermediate animals with the greatest certainty, only the first 21 animals found before the first smaller inflection point of the RHS curve were sequenced as resistant animals.

### DNA extraction and sequencing

DNA was salted out (Miller et al. 1987) from EDTA blood to produce high-quality DNA. DNA concentration was initially measured with the Nanodrop 2000 spectrophotometer (Thermofisher, USA) and diluted to 20–30 ng/µl. Before library preparation for next-generation sequencing, diluted DNA was quantified again with the Qubit 2 system (Thermofisher, USA) using the Qubit dsDNA broad range assay kit and the Qubit dsDNA high sensitivity assay kit. Subsequently, paired-end libraries were prepared with the TruSeq DNA PCR-free sample preparation kit (Illumina, USA). Generated libraries were quantified through qPCR with the Kapa Library Quantification Kit (Kapabiosystems, USA) and distribution of fragment sizes in the libraries was checked with the BioAnalyzer 2100 (Agilent Genomics, USA).

Whole genome sequencing was performed on the Illumina HiSeq2500 (Illumina, USA). Paired-end libraries (2 × 126 bp reads) were sequenced with a mean coverage of ten times (*n* = 48 animals) and four times (*n* = 10 animals), respectively.

### Computer analysis

Prior to further processing, raw data were quality checked for overrepresented and duplicate sequences with FastQC (www.bioinformatics.babraham.ac.uk/projects/fastqc). Duplicates were removed by picardtool and samtools (Broad Institute).

All programs used in further processing raw reads were embedded in Python scripts to connect the different steps and programs.

First, raw sequences were converted from a base call file (.*bcl*) to .*fastq* files and mixed probes were demultiplexed through the program bcl2fastq Conversion Software from Illumina (https://emea.support.illumina.com/downloads/bcl2fastq_conversion_software_184.html?langsel=/de/).

Next, sequences in .*fastq* format were aligned to the swine genome (*S. scrofa* 11.1, Genebank assembly accession: GCA_000003025.6) with the *mem* algorithm implemented in the Burrows Wheeler Alignment software (Li and Durbin 2009) and stored as .*bam* files. Quality control for the aligned sequences was performed by identifying read groups and tagging duplicated reads. Statistics were created with the utility flagstats from SAMtools (Li et al. 2009).

### Variant calling

Variants were called according to the best practices workflow from the Genome Analysis Toolkit (GATK) (DePristo et al. 2011). Quality scores such as the *phred* score created by sequencing systems are often prone to errors, which need to be corrected. Therefore, the base quality score recalibration (BQSR) implemented in GATK (DePristo et al. 2011; Van der Auwera et al. 2013) was applied. The program builds a model of covariation where known variants deposited in the dbSNP database are masked out in the input data. Remaining mismatches with the reference genome are then marked as errors and base quality scores are readjusted producing a new .*bam* file.

Next, HaplotypeCaller (DePristo et al. 2011; McKenna et al. 2010; Van der Auwera et al. 2013) of GATK was used to reevaluate the previously recalibrated variants. The program defines possible variant sites and uses De Bruijn-like graphs to reassemble all possible haplotypes. Implementing the Smith–Waterman algorithm, the program realigns the possible haplotypes against the reference haplotype to identify all possible variant sites. Using the PairHMM algorithm, the likelihoods for each previously called read are calculated and, by applying Bayes’ theorem, the most likely genotype is chosen for each sample. Finally, the variants are jointly called with the GenotypeGVCF to generate gVCFs (genomic variant calling files).

### Quality control

To further improve the quality of the variant call set, several hard filters from GATK were applied (Van der Auwera et al. 2013; DePristo et al. 2011; McKenna et al. 2010). The following parameters were considered: (i) the quality by depth (QualByDepth, QD), which is the quality score (Qual) divided by the sequencing depth (Depth); (ii) the phred-scaled *p* value using a Fisher-Exact test to detect strand bias (FisherStrand, FS); (iii) the root mean square of the mapping quality of reads across samples (RMSMappingQuality, MQ); (iv) the Mann–Whitney–Wilcoxon rank sum test for mapping qualities of reference and alternate reads (MappingQualityRankSumTest, MQRanksum); and (v) the Mann–Whitney–Wilcoxon rank sum test for the distance of the variant alleles from the end of the read (ReadPosRankSumTest, ReadPosRankSum). All the aforementioned filters were applied to SNPs and INDELs with different parameters (SNPs: QD < 2.0; FS > 60.0; MQ < 40.0; MQRanksum < − 12.5; ReadPosRankSum < − 8.0 and for Indels: QD < 2.0; FS > 200.0; ReadPosRankSum < − 20.0). Furthermore, variants with a general quality score (Qual) > 999, genotypes with a read depth < 4, multiallelic variants, samples having only ref/ref genotypes and samples with no genotype called were discarded.

### Variant effect prediction

The previously called variants were annotated using the variant effect predictor (VEP) from Ensemble (McLaren et al. 2016). The program identifies genes, transcripts, protein sequences, and regulatory regions potentially affected by the variants and outputs the location of the variants in base pairs and potential consequences on the protein sequence. From known variants, the matching reference sequence (rs) number is given based on the *Sus scrofa* 11.1 assembly.

### Genotype imputation

Due to the low coverage approach, imputation was necessary to ensure sufficient quality of the generated genotypes. Therefore, sequences of a population of 46 German Landrace animals, 46 Piétrain animals, 4 Hampshire animals as well as the newly sequenced 58 were used to replace missing data with known genotypes from the reference data. Therefore, we set all variants with a coverage of < 4 to missing and imputed the missing genotypes. Data were imputed using Beagle 4.1 using 15 iterations to estimate genotype phase (Browning and Browning 2007, 2016). We tested the robustness of the imputation based on SSC17, encompassing 63,494,081 bases (2.7% of Sscrofa11.1 genome). From a total of 1,180,160 variants called, 1,100,933 passed the filter and 456,550 had no missing genotypes. Out of these 456,550 variants with no missing genotypes, 2000 were randomly selected. Every second of the 2000 variants of 154 animals was set missing and imputed with Beagle version 4.1 with 15 iterations.

The mean concordance of true and imputed genotypes as a measure for the imputation accuracy was calculated as 0.9786. Thus, the imputation can be considered robust.

### Identification of candidate genes

All SNPs significant after FRD-BH correction in GWAS (Supplemental Table 1) were listed. As individual or minimal SNPs within a region imply random associations in a GWAS, these localizations were not further considered (SSC4, 16, 18, X). Two regions remained on SSC2, one on SSC12 and a larger region on SSC15. Subsequently, all SNPs were tested for their functionality based on their sequence. We tried to identify non-synonymous gene variants, then SNPs in the coding region of genes, in introns and finally in the vicinity of genes in the region of significant SNPs in decreasing order. The genes around the peak of the Manhattan plot were tested for their homolog-physiological suitability as candidate genes for resistance to *A. pleuropneumoniae*. One SNP from each region of chromosomes 2, 12 and 15 that was as close as possible to or within a homolog-physiological candidate gene was validated via Sanger sequencing or KASP genotyping. The GWAS was then repeated with the corrected genotypes and the significance of the validated markers was checked against the existing FDR-BH significance threshold. The four validated SNPs with the highest association to the phenotype (RHS) were used to calculate the effects of these SNPs and the explained variance. The individual steps of this procedure are described in detail below.

### Genome-Wide Association Study (GWAS)

The GWAS was mostly performed using Fisher’s exact test implemented in the program PLINK (Purcell et al. 2007). Fisher’s exact test is a variation of the chi-squared test where contingency tables are used to determine a possible difference between the expected frequencies of variants between cases and controls and the observed frequencies. Fisher’s exact test is a fixed-effect model. We then calculated association with the Cochran–Armitage test for trend and compared values with the chi-squared values to check for the genomic index. Only variants with a minor allele frequency > 0.05, a *p* value > 0.001 for Hardy–Weinberg equilibrium and a missing genotyping rate < 0.1 were included in the GWAS. A quantile–quantile (QQ) plot demonstrated possible genomic inflation due to population stratification and/or cryptic relatedness. Manhattan plots illustrated strength of the association between an SNP and a phenotype.

Variants in linkage disequilibrium were depicted using the program Haploview (Barrett et al. 2005). Significantly disease-associated SNPs were validated through Sanger sequencing (Sanger and Coulson 1975) and Kompetitive allele-specific PCR (KASP™, LGC Genomics, Teddington, Middlesex, UK).

Further statistics were generated using the Wald chi-squared test (Wald 1943), a test for the independence of two variables based on the difference between the observed and expected values and one-way ANOVA implemented in the SPSS statistics program (IBM).

## Results

### Phenotypes

All animals used in the present study were clinically, sonographically and radiographically healthy before infection. The pigs tested negative in antibody tests for *A. pleuropneumoniae*, *Haemophilus parasuis*, PRRSV and Influenza A. None of the pigs had an elevated rectal temperature or signs of dyspnea or respiratory distress.

After infection, individual scores were calculated for each day and totaled on day 7 p.i. to get the final scores. Pigs which died or were euthanized (*n* = 36) based on defined exit criteria before day 7 p.i. were assigned maximum score values. Scores encompassed the complete spectrum of values (Table 1) and were normally distributed. Sorting RHS values showed a steady increase in values until an RHS score of about 60. Thereafter, RHS values increased abruptly until they were consistently high (85–100) (Fig. 1).

**Table 1:** Clinical, pathological and microbiological scores of the 163 infected pigs (see Hoeltig et al. 2009 for calculation of scores)

Trait	Mean	SD	Minimum	Maximum	Lower CI (95%)	Upper CI (95%)
Clinical Score	8.51	10.59	0	35	6.88	10.14
RoeS	27.53	16.74	0	50	24.96	30.10
SoS	96.91	78.3	0	200	84.89	108.93
RHS	42.61	31.28	0.33	100	37.81	47.41
LLS	14.39	10.93	0	35	12.71	16.06
Mortality	0.22	0.41	0	1	0.16	0.28
ReIsoL	1.74	0.95	0	3	1.59	1.88

*CI* confidence interval, *RoeS* Roentgen Score, *SoS* Sonographic Score, *RHS* Respiratory Health Score, *LLS* Lung Lesion Score, *Mortality* Mortality before day 7 p.i., *ReIsoL* Reisolation Score

Pearson correlation demonstrated that all scores are highly correlated (Table 2) with *p* values ≤ 0.001. Most striking was the correlation between the RHS score and the other scores which ranged from 0.72 to 0.92. The scores contributed between 52.3 and 85.4 to the RHS.

**Table 2:** Correlations (Pearson Correlation) between clinical, pathological and microbiological scores in the 163 infected pigs

	Mortality	Clinical Score	RoeS	SoS	LLS	ReIsol
Clinical Score	0.96					
RoeS	0.72	0.75				
SoS	0.70	0.74	0.75			
LLS	0.70	0.70	0.75	0.73		
ReIsol	0.51	0.57	0.70	0.70	0.63	
RHS	0.86	0.90	0.91	0.92	0.80	0.72
RHS contribution rate (%)	73.4	80.6	82.8	85.4	63.6	52.3

All correlations have a *p* value ≤ 0.001

*RoeS* radiographic score, *SoS* sonographic score, *LLS* lung lesion score, *ReIsol* reisolation score, *RHS* respiratory health score

For this reason, the RHS score was selected as the phenotype to be included in all further analyses.

Based on RHS score values (Fig. 1), pigs were classified into ‘resistant’ (*n* = 21), ‘intermediate’ (*n* = 105), and ‘susceptible’ (*n* = 37) groups (Table 3). Mean values between groups were significantly different for all traits at *p* ≤ 0.001. Only mortality did not differ significantly between “intermediate” and “resistant” pigs.

**Table 3:** Classification of the pigs in resistant, intermediate and susceptible animals and the corresponding score values

Trait	Resistant (*n* = 21)		Intermediate (*n* = 105)		Susceptible (*n* = 37)	
	Mean	SE	Mean	SE	Mean	SE
Clinical Score	0.95	0.08	4.87	0.64	23.14	1.55
RoeS	4.48	0.4	24.81	1.2	48.32	1.1
SoS	2.43	0.63	82.81	6.32	190.54	4.34
RHS	4.3	0.28	34.97	2	86.02	2.5
LLS	1.29	0.31	11.34	0.63	30.47	0.8
ReIsol	0.27	0.05	1.73	0.07	2.59	0.1
Mortality	0	0	0.05	0.02	0.84	0.06

*RoeS* radiographic score, *SoS* sonographic score, *LLS* Lung lesion score, *ReIsol* reisolation score, *RHS* respirator

### Next-Generation Sequencing

Animals in the ‘resistant’ (*n* = 21) and ‘susceptible’ groups (*n* = 37) were sequenced. The animals were sequenced with a mean coverage of 10 ×.

Initially, 25,720,000 variants were detected from which 15,478,704 variants remained after quality control (see “Materials and methods”) and were included into the GWAS.

### Genome-Wide Association Study

A genome-wide association study (GWAS) was performed as a case–control study, applying ‘resistant’ and ‘susceptible’ as the two possible phenotypes. *p* values for all variants were corrected using FDR-BH (Supplemental Table 1).

**Fig. 2:**
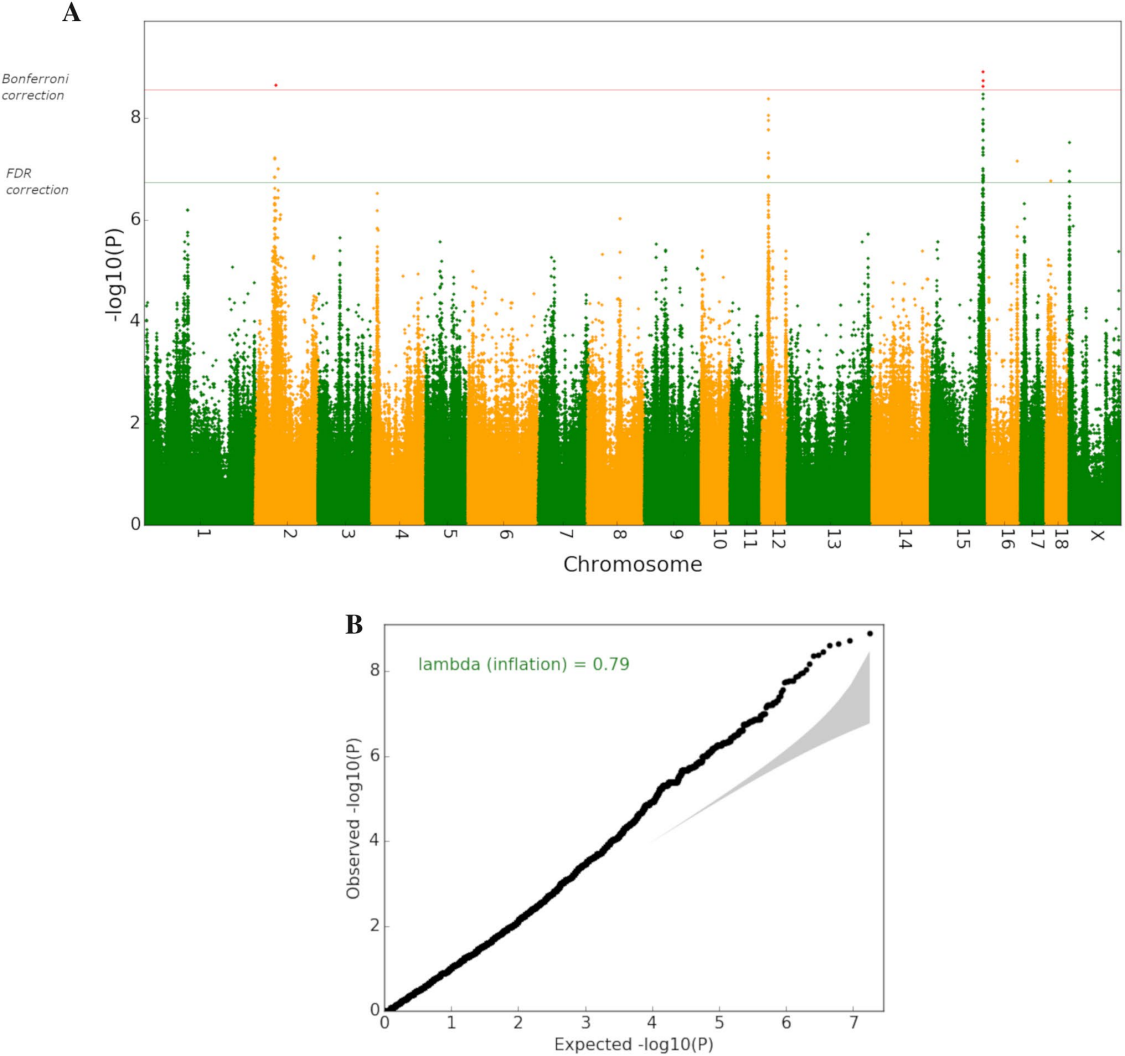
Manhattan plot with thresholds for Bonferroni correction and False Discovery Rate after Benjamini and Hochberg(A) and QQ plot of GWAS with the genomic inflation value lambda(B) showing the expected *p* value (-log) on the *x*-axis and the observed *p* value (-log) on the *y*-axis; (created with Python Pandas)

The GWAS revealed significantly associated variants mainly on chromosome 2, 12 and 15 (Fig. 2a, Table 4 and Supplemental Table 1), which were annotated with the variant effect predictor from ensemble. Singular variants on chromosomes 4, 16, 18 and X were also significantly associated. Because of their singularity, they were excluded from further analysis.

**Table 4:** Association of the different markers with the RHS score

Marker (SSC_position)	Method	Wald chi-squared test	*p* Value	ANOVA *p* value	Explained variance (%)
2_45022788	Sanger	36.29	1.32E−08	< 0.001	32.9
12_14641621	Sanger	18.34	1.34E−03	< 0.001	19.9
15_128476618	Kasp	16.77	5.54E−04	< 0.001	18.5
Combined markers		77.78	1.09E−11	< 0.0001	52.8

*Marker* the chromosome and position of the SNP, *Method* the method used to validate the SNP

The QQ plot and the genomic inflation factor (*λ* = 0.79) did not indicate population stratification and/or cryptic relatedness between animals (Fig. 2b).

Many variants were located in introns of genes (F-spondin gene (SPON-1) on SSC2, the platelet and endothelial cell adhesion molecule 1 gene (PECAM1) on SSC12, the collagen IV gene (COL4A4) and the rhomboid domain-containing protein 1 gene (RHBDD1) on SSC15), while some were located in intergenic regions (Supplemental Table 1). Significant SNPs on SSC2, 12 and 15 spanned 9.1 Mb, 4.7 kb and 3.3 Mb, respectively. SNPs on SSC12 spanned the PECAM1 gene, and SNPs on SSC15 spanned the RHBDD gene (spanning 113 kb) and the COL4A4 gene (spanning 130 kb). The SNP on SSC15 (rs339180611) was the only significant coding variant. As a 3 prime UTR variant, it is located in the coding region of the alpha chain of the collagen IV gene (COL4A4).

In the three regions with genomically significant SNPs, nine SNPs were selected and validated using Sanger sequencing or KASP genotyping. The selection was made with the program Haploview (Barrett et al. 2005) using default parameters. The SNPs had to be in linkage disequilibrium with the most significant SNP of the region while simultaneously within range of one of the functional candidate genes listed above (Supplemental Table 1). The different validated genotypes corresponded up to 95% with the genotypes generated by NGS. In some variants, many heterozygous variants were miscalled by the NGS system. After validation, GWAS was redone with the improved genotypes. Table 4 and Supplemental Table 1 show the results of the association redone with the validated genotypes.

**Fig. 3:**
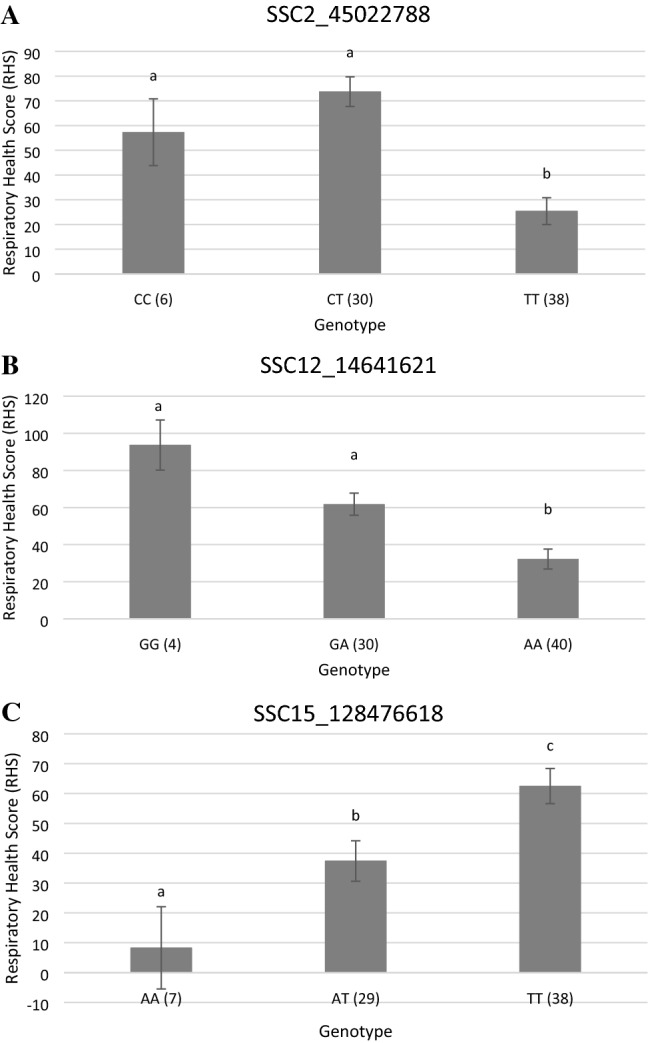
Means and standard deviations (SD) of RHS depending on the genotypes of the major SNPs on SSC2 (**a**), SSC12 (**b**) and SSC15 (**c**). Genotypes with different superscripts differ significantly with *p* < 0.05. The number of cases is given in brackets together with the genotypes (created with ms excel). Residuals were normally distributed

The three validated variants which had the highest correlation to the RHS score were used to calculate explained phenotypic variances (Table 4). SNP 12_14641621 on SSC12 explained up to 19.9%, SNP 15_128476618 on SSC15 up to 18.5% and SNP 2_45022788 on SSC2 up to 33% of the phenotypic variance of the RHS score (Table 4). When all three SNPs were calculated as a combined effect, they explained 52.8% of the phenotypic variance and were highly correlated to the RHS score (*p* = 1.09E−11). Means and standard deviations of RHS depending on the genotypes of the three major SNPs are given in Fig. 3.

## Discussion

As expected from several studies on the high susceptibility of pigs of the German Landrace breed to *Actinobacillus pleuropneumoniae* (Sørensen et al. 2011; Reiner et al. 2014a, b; Hoeltig et al. 2009), in the present study 36 of the infected pigs died or had to be euthanized before day 7 of the infection trial. Thirty-seven of the infected pigs exhibited RHS values below 10 or higher than 90. This shows a considerable range of sensitivity within the tested breed. Although serotype (ST) 7 (*AP76*) seems to be less pathogenic than serotype 2 in general (Jacobsen et al. 1996; Maas et al. 2006a), this strain was selected because it causes severe herd problems in practice (Gottschalk and Lacouture 2015). Serotypes allow only a limited assessment of the expected virulence, but ST7 is generally classified as clearly virulent (Frey 2003) alongside ST2. For the study, it was less important to use the strain with the highest virulence than to use a strain that would lead to segregation of the level of resistance in the population studied. This goal was successfully achieved with the ST 7 strain in our preliminary work studies (Reiner et al. 2014a, b). This strain can serve as an example, and further research should test for differing effects with other strains. Our preliminary studies (Hoeltig et al. 2009) showed clear differences in resistance between different pig populations. The population of the German Landrace used in this study showed the strongest segregation of sensitive, intermediate and resistant phenotypes. With the help of the selected *Actinobacillus pleuropneumoniae* strain and the Landrace population used in the present study, QTL for the resistance of pigs to pleuropneumonia has already been mapped (Reiner et al. 2014a). Therefore, it seemed to be consequent to include the same strain and the same breed for a fine-mapping approach. Further studies are necessary to verify the general validity of the results.

Up to now, information on genetic resistance to pleuropneumonia has only been available as quantitative trait loci (QTL) mapped on the basis of microsatellite markers (Hoeltig et al. 2009; Gregersen et al. 2010; Sørensen et al. 2011; Reiner et al. 2014a, b). Gregersen et al. (2010) found six QTL on SSC 2, 8, 12, 13, 14 and 18 in a study with 7470 animals, significant at a chromosome-wide level, when screening for dorso-caudal chronic pleuritis in slaughter pigs.

Reiner et al. (2014a) mapped 7 QTL for resistance to pleuropneumonia to SSC 2, 6, 12, 13, 16, 17 and 18 in a F2 crossbred family (Hampshire × German Landrace). The QTL were significant on at least a chromosome-wide level. The QTL on SSC2, 12 and 18 corresponded between both studies. However, the QTL region on SSC2 from the study by Reiner et al. (2014a) clearly differed from the peak region in the present study. The center of the QTL can be estimated at position 145 MB on SSC2, with a relatively small range of 6.6 MB. The centers of the peaks on SSC2 in the present study were at position 49.5 MB, with a relatively wide range of about 9 MB. However, this region might contain more than one candidate gene as there are two narrow centers of significant SNPs, one at around 45 MB and the other at around 54 MB of the chromosome. Searching for candidate genes in this chromosomal region was difficult because larger areas are still incompletely annotated here. This is because SNPs from the 45 MB region overlap with the SPON-1 gene. This might be a valuable positional candidate gene—and because of its role in *A. pleuropneumoniae* disease (see below) also a homolog-physiological candidate gene—for further studies.

The QTL on SSC12 (Reiner et al. 2014a) peaked between 5 and 63 MB of the chromosome with a confidence interval of around 78 MB. The peak of the SNPs in the present study mapped to position 14 MB on SSC12 with a range of only 4.7 kB and with PECAM-1 as the positional and homolog-physiological candidate gene. The study by Reiner et al. (2014a) did not map a QTL to SSC15. In the present study, there was a wide range of significant peaks around region 128 MB spanning 3.3 Mb. These SNPs map to the region of two interesting candidate genes: RHBDD and COL4A4. Both warrant investigation in more detail in future studies.

The present study used the same German Landrace population as the study by Reiner et al. (2014a), with the aim of confirming the QTL results and finding hints to the genes involved in a fine-mapping approach. To achieve this goal, the study level was moved from the F2 family to a segregating, commercial population to use more informative meioses. In addition, the 155 gene markers from the QTL study were replaced by more than 15 million markers generated by genotyping by sequencing. The distribution of phenotypes suggests a polygenic inheritance with the participation of a few major genes. This assumption is also confirmed by a comparison with literature data (Hoeltig et al. 2009; Xie and Muller 1993; Newton et al. 1997; Sun et al. 1996; Liao et al 1997; Maas et al. 2005; van Kuppelvelt et al. 1995) and the ‘common disease-common variants’ hypothesis describes how common diseases are caused or influenced by many common variants in humans and some of those variants may cause susceptibility or resistance against complex diseases (van Kuppelvelt et al. 1995).

It was thus clear that the genes sought would explain significantly less than 100% of the phenotypic variance. Intermediate phenotypes should have a larger, heterozygous mixture of different gene variants that would disguise the effects of the major genes.

To maximize the differences between animals, we tried to exclude intermediate phenotypes as far as possible. This was easy to implement for sensitive animals, because between RHS 60 and 90 there was an abrupt deterioration of the resistance situation in the investigated animals. In contrast, there were only minor differences in the increase of RHS in the transition between animals with the most pronounced resistance and intermediate phenotypes. Therefore, only the 21 animals with the lowest RHS were included, which were responsible for the flattest slope of the RHS curve.

With this approach, it was possible to confirm effects on SSC2 and SSC12. No references to the QTL on SSC18 were found. Instead, a further effect on SSC15 was discovered, which was neither found in the work of Gregersen et al. (2010) nor in our earlier work (Reiner et al. 2014a, b). The differences between the studies indicate that gene variants responsible for differences in resistance between two populations do not necessarily play the same role within populations. It is conceivable that these variants do not sufficiently segregate within a breed, if at all. Clarifying such relationships is an important task for future studies.

Thus, it can be assumed that restricting the animals to the extremes made it possible to identify the candidate genes on SSC2, SSC12 and SSC15. At the same time, however, we can suppose that far more genes might be involved in resistance/sensitivity to pleuropneumonia. These genes should explain lower percentages of variance and elude identification using the marker approach of the present study.

Compared to a QTL study by Gregersen et al. (2010), our preliminary study (Reiner et al. 2014a, b) clearly showed that the increased accuracy of the phenotypes that can be achieved under the given experimental infection and phenotyping conditions (which were also applied in the present study) could save considerable numbers of experimental animals. However, despite the accurate and detailed individual animal infection and phenotyping, the significance of the results could certainly have been increased by using more animals, which should be a goal for further studies. Due to the normal distribution of resistance/sensitivity to *A. pleuropneumoniae*, the number of experimental animals should be increased considerably in order to obtain more animals of the extremes for comparison. This was not possible in the present study for animal welfare reasons and because of the high experimental demands on infection and phenotyping.

**Fig. 4:**
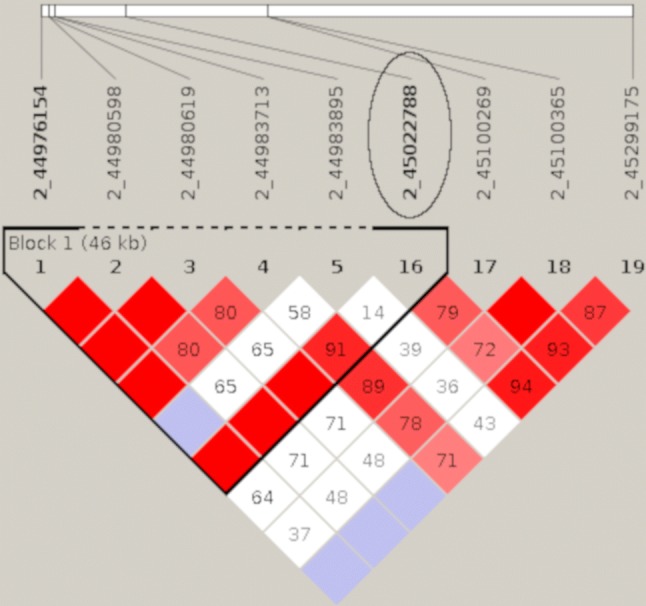
A Linkage Disequilibrium (LD) plot for the SPON1 gene. The encircled variant (2_45022788) represents the variant on SSC2 which is significantly associated with the RHS score. Numbers in the squares depict the D’ value (multiplied by 100). Red squares without a number have a D’ value of 1. Bold printed variants build up a linkage block suggested by the program Haploview

**Fig. 5:**
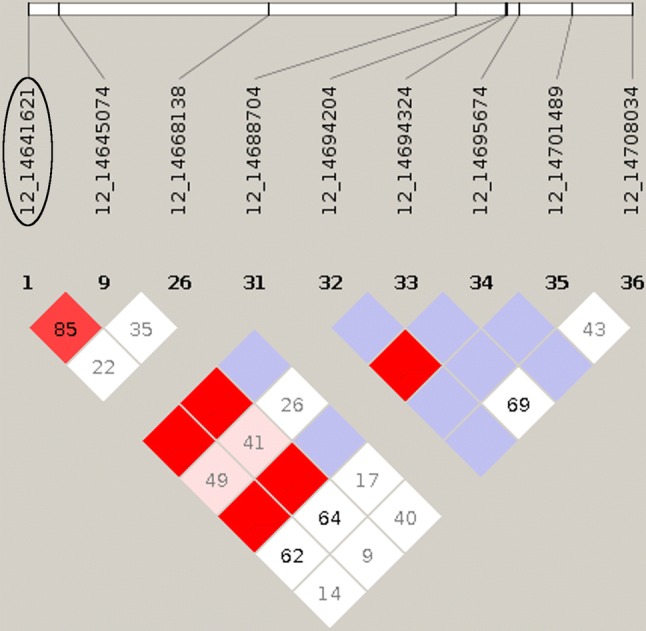
A linkage disequilibrium (LD) plot for the PECAM1(CD31) gene. The encircled variant (12_14641621) represents the variant on SSC12, which is significantly associated with the RHS score. Numbers in the squares depict the D’ value (multiplied by 100). Red squares without a number have a D’ value of 1

**Fig. 6:**
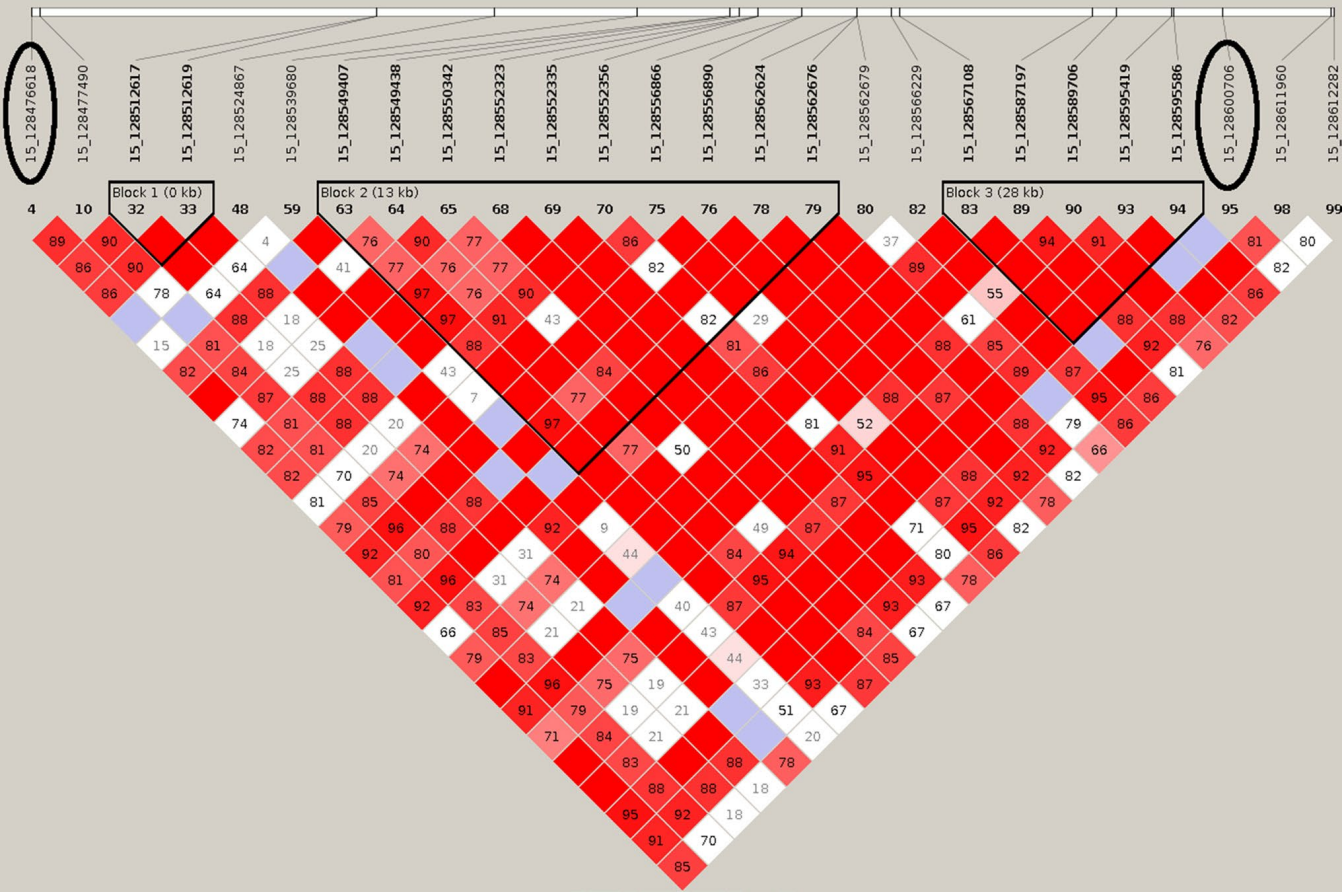
A linkage disequilibrium (LD) plot for the COL4A4 gene. The encircled variants (15_128476618 and 15_128600706) represent variants on SSC15 which are significantly associated with the RHS score (Supplemental Table 1). Numbers in the squares depict the D’ value (multiplied by 100). Red squares without a number have a D’ value of 1. Bold printed variants build up a linkage block suggested by the program Haploview

Error-free DNA sequences cannot be generated with either fourfold or tenfold coverage. However, the aim of the present study was not to generate absolutely reliable and complete sequences, but a high marker density for GBS (genotyping by sequencing). Most of the discovered variants were intron or intergenic variants. Only rs339180611 as a 3 prime UTR variant is located in a coding region of the alpha chain of the collagen IV gene (COL4A4) (Supplemental Table 1). An analysis of the genetic linkage between the validated intron variants and all coding variants of the affected genes showed high linkage disequilibrium between the genotyped variants and synonymous SNPs on SSC2 and SSC12 and a high linkage disequilibrium between rs339180611 and several variants in 5 prime UTR locations as well as in splice regions on SSC15 (Fig. 4, 5, 6).

The variants on Chr. 2 explained 32.9% of the phenotypic variance, with a tendency, but not a significant overdominance effect that can be found in several linked SNPs within this region. The phenomenon of overdominance is well known from the polar overdominance of an SNP between the DLK1 (delta-like-1 homolog [Drosophila] and the MEG3 (maternally expressed gene 3), a non-protein coding gene from callipyge sheep (Cockett et al. 1996; Schulze-Koops et al. 1992) that causes an upregulation of the parent-of-origin allele-specific genes (Bidwell et al. 2014) and results in greater muscle mass in sheep with the heterozygous genotype.

The significant variants on Chromosome 2 were mostly intron or intergenic variants, but some lay in the f-spondin gene. The f-spondin or SPONDIN-1 gene is described as an extracellular matrix protein and adhesion molecule in vertebrates that attaches to the embryonic floor plate and promotes axonal outgrowth (Lee et al. 2016). It has also been described as promoting the release of cytokines such as interleukin 6 as an inflammatory response, especially as a result of LPS stimulation of vascular smooth muscle cells (Lee et al. 2016) in mice. Bruun et al. (2012) were able to demonstrate an upregulation in matrix metalloproteinase 9 and 12 in porcine lung infections, whereas matrix metalloproteinases often work as the first barrier in infections with gram-negative bacteria, and SPONDIN-1 can supposedly mediate matrix metalloproteinase 9 expression (Lee et al. 2016). As LPS is one of the main virulence factors of *A. pleuropneumoniae*, triggering a key response of the host cell defense (Cho et al. 2005) and introducing endothelial and vascular damages to lung tissue, variants in SPONDIN-1 could play a significant part in host defense against the pathogen.

It is questionable what role the identified SNPs could play. Only synonymous mutations were detected in the coding sequence. However, as intron variants, even synonymous variants might show effects on mRNA-splicing, mRNA-stability, mRNA-structure and protein folding (Hunt et al. 2009; Wang et al. 2015). On the other hand, it cannot be ruled out that a linked, functional variant remained undiscovered during sequencing due to the low coverage. Further studies are needed to provide more insight into the role of SPONDIN-1 polymorphisms in resistance/susceptibility to pleuropneumonia.

The SNP on SSC12 (Supplemental Table 1) is an intron variant of the platelet and endothelial cell adhesion molecule 1 gene (PECAM 1). It explained 19.9% of phenotypic variance. PECAM 1 (CD31) belongs to the ig-immunoreceptor tyrosine-based inhibitory motif (ITBIM) superfamily that regulates hematopoietic cell function, leukocyte transmigration, immune homeostasis, thrombosis, suppression of apoptotic cell death and vascular permeability (Xie and Muller 1993; Newton et al. 1997; Sun et al. 1996; Liao et al. 1997). Maas et al. (2005) used PECAM 1-deficient mice to prove the PECAM1 function in LPS-induced endotoxin shock reactions. PECAM 1-deficient mice were more susceptible to LPS than mice with an intact CD31 receptor. Lovelace et al. (2013) found that PECAM 1-deficient mice are more resistant to infection with *Salmonella typhimurium* than wild-type mice. Analogous to the variants on SSC2, the role of PECAM 1 in porcine pleuropneumonia needs to be addressed by further studies.

The variants on SSC15 (Table 3) cover the region of COL4A4. They explained up to 18.5% of phenotypic variance. Collagen makes up more than 60% of connective tissue in the human lung (Van Kuppevelt et al. 1995). The protein plays an important role in the attachment of pathogenic and commensal bacteria through specialized adhesins (Mukai et al. 1997; Schulze-Koops et al. 1992; Sebghati et al. 1998; Switalski et al. 1993; Trust et al. 1991; Westerlund and Korhonen 1993). The adhesion of *A. actinomycetemcomitans* to human collagen through surface proteins has been reported (Mintz and Fives-Taylor 1999). Enríquez-Verdugo et al. (2004) were able to demonstrate that *A. pleuropneumoniae* serotype 1 can adhere to collagen I, III, IV and V in swine through a 60 kDA outer membrane protein.

Both the intron variant and the 3 prime UTR variant show genetic linkage to splicing-region variants and 5 prime UTR variants of the gene. Thus, altered splicing sites affecting protein function are possible. In particular, the 5 prime and 3 prime SNPs have the potential to change and interfere with the translational process of a protein through structural and functional change in the corresponding mRNA (Chatterjee and Pal 2009). Therefore, the variants on chromosome 15 might be part of a molecular mechanism that restricts *A. pleuropneumoniae* from adhesion to the extracellular matrix of the lungs of affected swine. Thus, collagen genes are further interesting starting points for future research into pathogenesis and resistance to pleuropneumonia in swine.

## Conclusion

The present study confirms effects on SSC2 and SSC12 for resistance/sensitivity to pig pleuropneumonia and maps a further effect on SSC15. By increasing the marker density and the number of informative meioses, a fine mapping down to the level of possible candidate genes was achieved. These genes are functionally related to the pathogenesis of pleuropneumonia. Gene markers were derived from the identified SNPs, which individually explain 15%, 18% and 33% of the variance, respectively. However, a limited number of animals, limited sequencing coverage and gaps in the reference genome prevented the identification of functional gene variants. This task is reserved for future studies.

## Electronic supplementary material

Below is the link to the electronic supplementary material.

Supplementary material 1 (XLSX 22 kb)
